# Natural Shikonin Potentially Alters Intestinal Flora to Alleviate Acute Inflammation

**DOI:** 10.3390/microorganisms11092139

**Published:** 2023-08-23

**Authors:** Ying Liang, Dongen Ju, Wenna Liu, Dan Wu, Yujia Zhao, Yaya Du, Xi Li, Minggao Zhao

**Affiliations:** 1 Precision Pharmacy & Drug Development Center, Department of Pharmacy, Tangdu Hospital, Fourth Military Medical University, Xi’an 710038, China; lynne3665@163.com (Y.L.); liuwennahx888@163.com (W.L.); lenglu0613@163.com (D.W.); 18762319358@163.com (Y.D.); lixi_seky@163.com (X.L.); 2 Department of Urology, Xijing Hospital, Fourth Military Medical University, Xi’an 710032, China; dn19951119@163.com; 3 Department of Oncology, The First Affiliated Hospital, School of Medicine, Xi’an Jiaotong University, Xi’an 710086, China; zhaoyj0302@163.com

**Keywords:** shikonin, anti-inflammation, intestinal flora, 16S rDNA

## Abstract

Shikonin, derived from the herb Lithospermum erythrorhizon (Purple Cromwell), is extensively utilized in traditional Chinese medicine as an anti-inflammatory agent; however, its effect on the intestinal flora is not yet known. Herein, we demonstrate that, compared to a blank control group, the intragastric administration of shikonin suppressed the swelling rate of ears in a mouse model of acute inflammation in a dose-dependent manner via animal experiments; furthermore, the 20 mg/kg shikonin treatment exhibited the highest inhibitory effect. In formal animal experimentation, we discovered that the inhibitory effect of shikonin with 20 mg/kg on inflammation was closely linked to the intestinal flora, whereby the microbiota phylum was altered in feces through a 16S rDNA sequencing analysis, implying that shikonin improves gut microbiota structures and compositions to counteract inflammation. Notably, using a real-time quantitative polymerase chain reaction (RT-qPCR), a Western blotting assay, and an immunohistochemistry (IHC) assay, we found that inflammatory cytokines such as TNF-α, IL-6, and IL-1β reduced in both the shikonin-administration group and the positive control group than those in the blank control group, as expected. To the best of our knowledge, this is the first study to outline the underlying mechanism through which shikonin acts on gut microbes to alleviate acute inflammation, providing an alternative mechanism for shikonin to become a preventive agent in countering inflammation.

## 1. Introduction

Undoubtedly, humans live along with a large amount of microbiota in their guts, especially bacteria. Recently, the gut microbiota has become a new area of focus in discerning the advancement and evolution of various diseases [1,2,3]. The gut microbiota, also recognized as the intestinal flora, is a group of microorganisms that inhabit the gastrointestinal tracts of humans, with about 100 trillion archaea and bacteria [4,5,6,7]. The human intestinal flora generally consists of two bacterial phyla: Firmicutes and Bacteroidetes [8,9]. Great progress has been made in identifying the diversity or composition of the intestinal flora. For instance, a decreased gut bacterial content is a known hallmark of chronic disease [10,11,12]. In addition, gut bacterial genera, such as Roseburia and Bifidobacterium, exhibit biological antagonism effects; yet, Streptococcus and Escherichia/Shigella are destructive to the gut mucosa [13]. However, the diversity and structure of the bacterial population can be affected by the host’s diet [14,15].

Traditional Chinese medicine (TCM) has been eminent in China and its surrounding areas for many years. It is widely used in East Asia and is a complementary medicine globally [16]. Therefore, TCM is anticipated to gain great ground in clinical treatment in the future, although certain questions about its uncertain molecular mechanisms remain unanswered [17,18]. Shikonin is a bioactive pigment extracted from “Zicao” in TCM. Shikonin exhibits anti-inflammatory effects in different models [19,20]. For example, shikonin treatment significantly suppressed the expression of two critical proteins, p-IκBα and p-p65, functioning in the NF-kB signaling pathway, which further decreased the levels of inflammatory factors, including IL-6, TNF-α, IFN-γ, and iNOS [20]. Moreover, shikonin exhibited an anti-proliferative effect on SK-BR-3 and MCF-7 breast cancer cells via G0/G1 arrest and apoptosis, indicating that it inhibited tumor proliferation and growth [21]. These findings suggest that shikonin can resist inflammation and suppress tumor development. However, shikonin cannot be widely applied in clinical treatment because of its unclear underlying mechanisms.

In fact, TCM usually exhibits its effects through the gut microbiota [22,23,24,25]. Studies have demonstrated that TCM can directly alter the diversity and composition of gut microbiota [23,26,27]. Some TCM can indirectly influence the composition of the microbiota by influencing the host’s immune system [14]. As mentioned above, TCM maintains the hemostasis of the gut microbiota and prevents destructive pathogens in healthy and diseased conditions. However, the crosstalk between shikonin and gut bacteria still needs to be characterized.

To understand the effect of shikonin in the intestinal flora, we undertook experiments using an acute mouse model induced with xylene. The intragastric administration of shikonin inhibited ear edema in this model by decreasing the expression levels of TNF-α, IL-6, and IL-1β via qPCR detection, Western blot, and IHC assays. Notably, the inhibitory effect was associated with intestinal flora alterations via 16S rDNA sequencing analysis. Furthermore, the LEfSe and MetaStat analyses highlighted that shikonin improves the composition and function of the gut flora in mouse models. Thus, our findings revealed that shikonin modulates the gut microbiota to exert anti-inflammatory effects, shedding new light on the mechanism through which shikonin helps to resist inflammation.

## 2. Results

### 2.1. Shikonin Alleviated Inflammatory Response in a Xylene-Induced Mouse Model of Acute Ear Swelling

**Figure 1:**
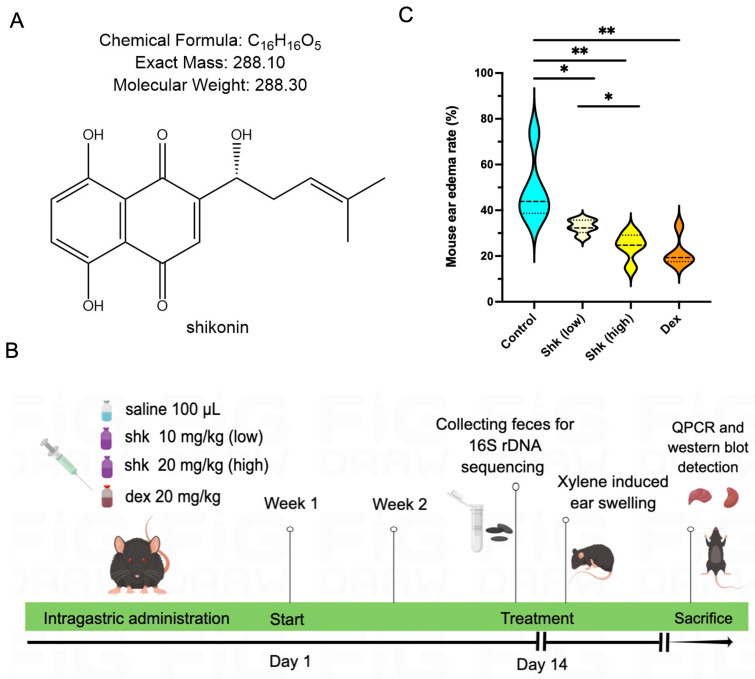
Shikonin inhibits xylene-induced acute ear edema in mice. (**A**) The chemical structure and formula of shikonin. (**B**) The study design of the in vivo preclinical experiment. Four groups of mice were intragastrically administered soybean oil (100 μL/each), dexamethasone (20 mg/kg), and low- and high-dose shikonin (10 and 20 mg/kg), respectively, for 14 days. On day 14, acute ear edema was established by administering xylene. The acute ear edema model was induced using xylene. The liver and kidney tissues were harvested to perform qPCR, Western blot, and immunohistochemical assays. Ctrl, shk, and dex represent soybean oil, shikonin, and dexamethasone, respectively. (**C**) Ear edema rates in the xylene-induced mouse model. The shikonin and dexamethasone treatment groups exhibit significantly lower ear edema rates than the control group, and high-dose shikonin exerts a more notable effect on the ear edema rate than low-dose shikonin. * indicates *p* < 0.05, ** indicates *p* < 0.01, *p* < 0.05 means significant difference.

Figure 1A presents the compound structure of shikonin, a component extracted from the traditional herb, Purple Cromwell. Initially, we aimed to establish the anti-inflammatory effect of shikonin, as well as to determine its most effective concentration. After establishing the xylene-induced ear edema model, we calculated the edema rates in control, shikonin (10 and 20 mg/kg dosages)-gastric administration, and dexamethasone-positive control groups in an animal assay. The shikonin concentration was based on a previous report [28,29]. Figure 1B illustrates the in vivo study design of the animal assay (prepared using Figdraw). As shown in Table 1, the treatment with shikonin dose-dependently suppressed ear swelling in the model mice (*p* < 0.05), in a manner that was similar to dexamethasone, the positive control. The inhibition rates in the low-dose shikonin (10 mg/kg), high-dose shikonin (20 mg/kg), and dexamethasone groups were 11.22, 11.34, and 9.10%, respectively. Moreover, the mean mouse ear edema weights were 11.81 ± 0.98, 6.94 ± 1.04, 5.61 ± 1.18, and 4.76 ± 0.73 mg in the control, low-dose shikonin, high-dose shikonin, and dexamethasone groups, respectively. The mouse ear swelling rates upon the topical application of shikonin (23.19%) and dexamethasone (22.36%) were significantly lower than that of the blank control group (34.60%), as shown in Figure 1C (*p* < 0.05). Thus, shikonin may potentially mitigate xylene-induced acute ear swelling in mice.

### 2.2. Shikonin Treatment Reduced the Diversity of Intestinal Flora in a Mouse Model of Acute Inflammation

**Figure 2:**
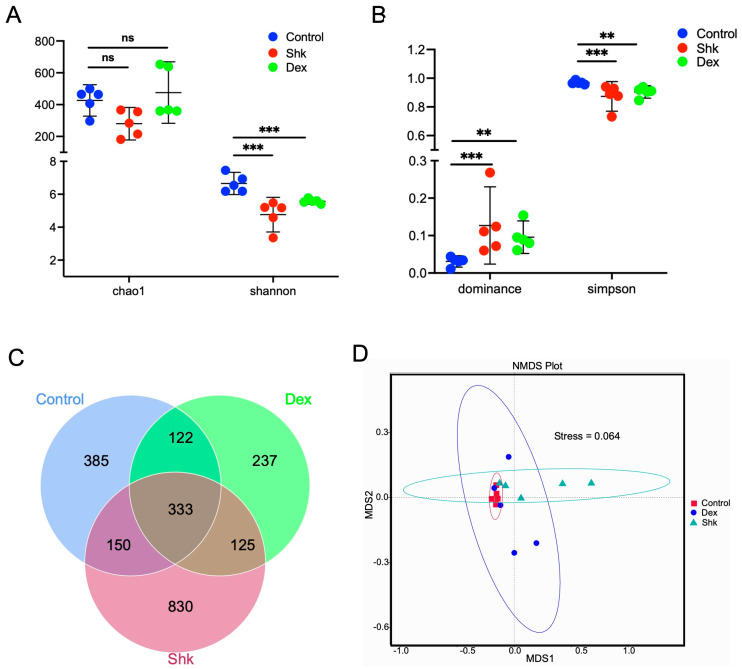
Shikonin treatment alters intestinal flora diversity. (**A**,**B**) Considering the alpha diversity, the shikonin and dexamethasone groups display a reduction in the Shannon index but not the Chao1 index (**A**), along with elevated Simpson and dominance indices compared with the control group (**B**). (**C**) The Venn diagram of three groups. (**D**) NMDS analysis of intestinal flora of the three groups. ** indicates *p* < 0.01, *** indicates *p* < 0.001, *p* < 0.05 means to be significant, and ns indicates no significance. Shk, shikonin; Dex, dexamethasone; NMDS, non-metric multidimensional scaling.

Certain intestinal flora have been closely associated with inflammatory molecules capable of inducing inflammation across the whole body. In the mouse model of acute ear edema, we found that 20 mg/kg shikonin exerted the highest anti-inflammatory effect; hence, this dose was used in subsequent experiments. We next explored the anti-inflammatory effects in the blank control, shikonin (20 mg/kg), and dexamethasone on intestinal flora by performing 16S rDNA (V3-V4 region) sequencing of the feces collected from each mouse group to identify alterations in intestinal microbiota after the shikonin intervention. After successfully constructing the genomic DNA library for the mouse feces of each group, 16S rDNA sequencing was performed to determine the effects of shikonin and dexamethasone (data accession number: PRJNA892764). As a result, we identified 2181 amplicon sequencing variants (ASVs) in samples. As shown in Figure 2A,B, the α diversity analysis revealed that treatment with 20 mg/kg shikonin or dexamethasone could significantly reduce the microbiota diversity, as demonstrated by Shannon and Simpson indices and dominance (*p* < 0.05); however, the species content (reflected by Chao1 index) was not notably altered. As shown in Figure 2C, the Venn diagram illustrated the presence of 333 overlapping ASVs among the control, shikonin, and dexamethasone groups, along with 385, 830, and 237 specific ASVs in the control, shikonin, and dexamethasone groups, respectively (Figure 2C). This indicates that the species composition of the intestinal flora was distinctive in each mouse group. To determine the beta diversity discrepancy of gut microflora among the three groups, non-metric multidimensional scaling (NMDS) analysis was performed based on unweighted UniFrac distance. As shown in Figure 2D, the NMDS results demonstrated evident variations in the microbiota profile of the three groups. 

Collectively, these results indicate that shikonin could alter the diversity of the gut microbiota in the experimental mouse model. Moreover, these findings suggest that the anti-inflammation role of shikonin on acute ear swelling in mice may be mediated via the intestinal flora.

### 2.3. Shikonin Treatment Altered Gut Microbiota Abundance in Mice

**Figure 3:**
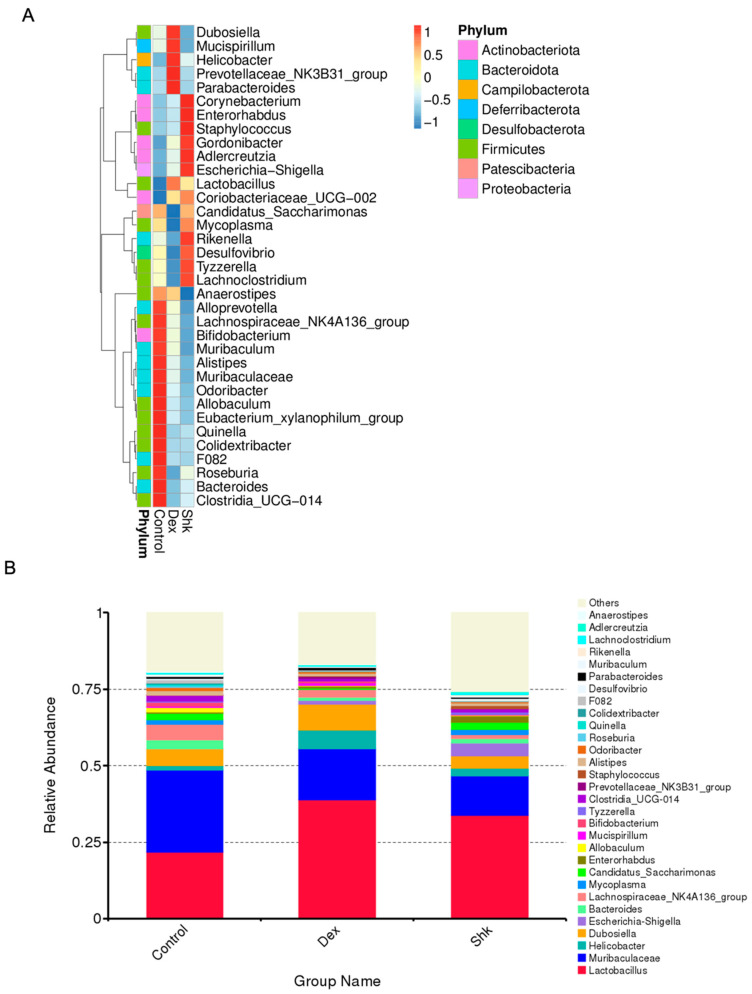
Shikonin treatment can alter the gut composition of mice. (**A**) Heatmap of the relative abundances of bacterial taxa in three groups. (**B**) The top 30 genera with the highest relative abundance in each group. Shk, shikonin; Dex, dexamethasone.

Next, we profiled the relative abundance to visualize ASVs in each group, as presented in the heatmap of taxa summaries (Figure 3A). Compared with the blank control group, the shikonin treatment group exhibited increased abundances of *Corynebacteroides*, *Enterorhabdus*, and *Adlercreutzia*, which belong to *the Actinomycetota* phylum. Conversely, shikonin treatment decreased abundances of the *Bacteroidota* phylum, including *Alloprevotella*, *Muribaculum*, *Alistipes*, *Muribaculaceae*, and *Bacteroides*. 

As shown in Figure 3B, the top 30 genera with the highest relative abundance were *Lactobacillus*, *Muribaculaceae*, *Helicobacter*, *Dubosiella*, *Escherichia-Shigella*, *Bacteroides*, and *Lachnospiraceae_NK4A136_group*, which were predominantly cataloged to the three most abundant phyla, i.e., *Firmicutes*, *Bacteroidetes*, and *Campliobacterota.* This was consistent with the findings of a previous report [30]. Compared with the control group, the shikonin and dexamethasone treatment groups displayed an increased abundance of *Lactobacillus, Helicobacter*, and *Escherichia-Shigella*, while that of *Muribaculaceae*, *Dubosiella*, *Bacteroides*, and *Lachnospiraceae_NK4A136_group* was decreased. Considering these microbes, the beneficial effects of an increased *Lactobacillus* abundance have been previously reported, consistent with our result [31]. Taken together, these results demonstrate that shikonin could modulate the microbiota composition.

### 2.4. Identification of Flora Exhibiting Significantly Altered Abundance Using Linear Discriminant Analysis (LDA)

**Figure 4:**
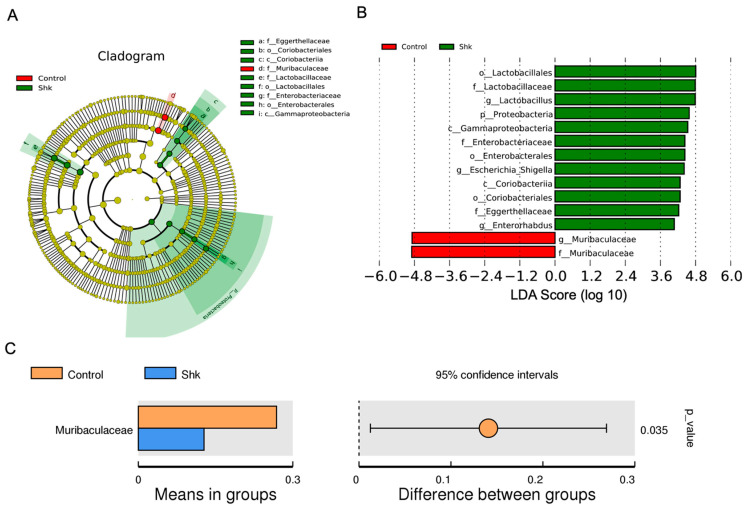
LEfSe analysis to identify specific bacterial taxa related to shikonin. (**A**) Cladogram indicating the phylogenetic distribution of microbiota correlated with the shikonin or control group. (**B**) LDA scores of the differently abundant microbiota in Cladogram. (**C**) Differences in abundance between the control and shikonin groups. *p* < 0.05 indicates significance. LEfSe, linear discriminant analysis effect size. Shk, shikonin.

To identify the specific bacterial taxa related to shikonin, we performed LDA effect size (LEfSe) to compare changes in species abundance between the control and shikonin groups. As shown in Figure 4A, we identified three bacteria species that were increased at the family level as key discriminants, including *Eggerthellaceae* and *Enterorhabdus* (*Actinobacteria*), *Lactobacillaceae* (*Firmicutes*), and *Gammaproteobacteria* (*Proteobacteria*) (all LDA scores (log10) > 3.6, Figure 4B), in the feces of shikonin-treated mice, whereas *Muribaculaceae* was the most abundant microbiota in the control group (LDA score (log10) > 4.8, Figure 4B). The *t*-test was used to analyze species differences between the control and shikonin groups. Consistent with our findings, a recent study has shown that the disruption of *Firmicutes* and *Actinobacteria* abundance could increase the occurrence of bacterial wilt disease in tomatoes, indicating that *Firmicutes* and *Actinobacteria* phyla exert a beneficial effect on disease suppression [32]. Based on our findings, only *Muribaculaceae* was significantly overrepresented in the blank control group at the genus level (Figure 4C). The data indicate that shikonin treatment could significantly alter the abundance of intestinal flora. 

### 2.5. Shikonin Treatment Decreased the Expression Levels of Inflammatory Factors Tumor Necrosis Factor (TNF)-α, Interleukin (IL)-6, and IL-1β in the Mouse Model

**Figure 5:**
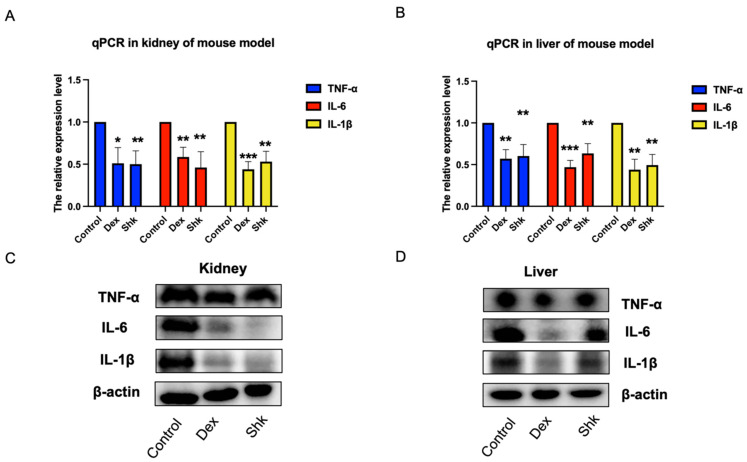
Shikonin reduces the expression levels of TNFα, IL-6, and IL-1β. (**A**,**B**) Shikonin and dexamethasone treatment reduce the expression levels of *Tnfα*, *Il-6,* and *Il-1β* in the kidney and liver tissues, as determined via qPCR. (**C**,**D**) Shikonin and dexamethasone treatment groups exhibit significantly lower TNF-α, IL-6, and IL-1β protein levels in the kidney and liver tissues than the control group, as determined via a Western blot assay (**C**). * means *p* < 0.05, ** indicates *p* < 0.01, *** indicates *p* < 0.001, *p* < 0.05 indicates a significant difference. IL-β, interleukin-β; IL-6, interleukin-6; TNF-α, tumor necrosis factor-α.

Given that inflammatory cytokines TNF-α, IL-6, and IL-1β are known to participate in the anti-inflammatory effects of herbs [33,34], we assessed mRNA and protein levels of the collected tissues from mouse models after shikonin treatment. We performed qPCR and Western blot assays to evaluate the effects of shikonin on inflammation. As shown in Figure 5A,B, the expression levels of *Tnfα*, *Il-6*, and *Il-1β* were significantly reduced in the kidney and liver tissues of the shikonin- and dexamethasone-treated mice when compared with those in the control mice. Furthermore, the shikonin and dexamethasone groups exhibited significantly lower TNFα, IL-6, and IL-1β protein levels in the kidney and liver tissues than the control group, as determined via Western blot (Figure 5C,D). Taken together, these results, validated by qPCR and Western blot assays, indicated that shikonin could exert anti-inflammatory properties similar to dexamethasone.

**Figure 6:**
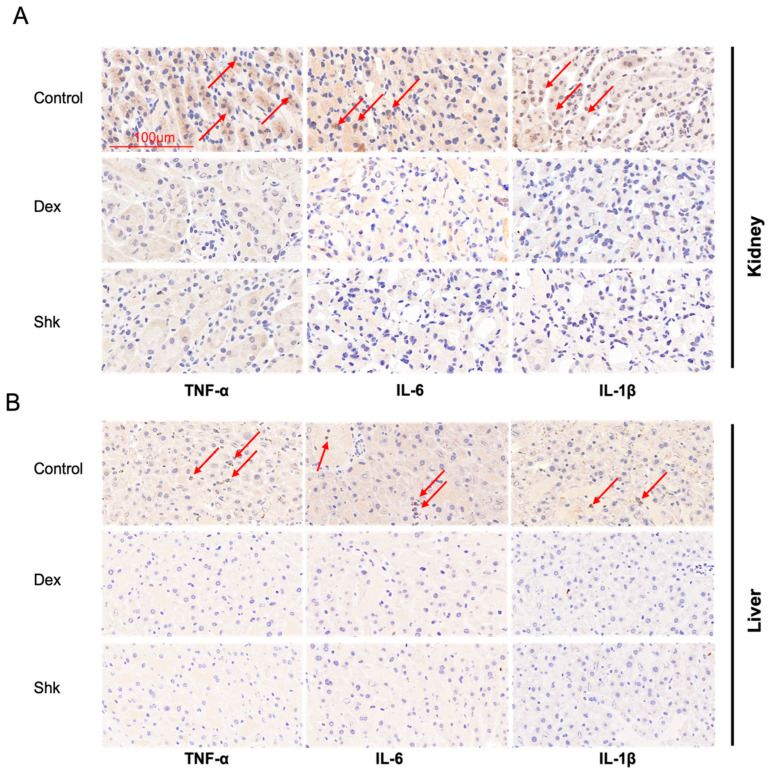
Immunohistochemical detections of TNF-α, IL-6, and IL-1β in the kidney and liver tissues. (**A**,**B**) Protein levels of TNF-α, IL-6 and IL-1β are notably lower in the kidney and liver tissues of the shikonin and dexamethasone groups than those in the control group. IL-β, interleukin-β; IL-6, interleukin-6; TNF-α, tumor necrosis factor-α. Red arrows show positive staining for the cytokines.

To evaluate the influence of shikonin on inflammation, we detected the alterations in cytokines TNFα, IL-6, and IL-1β by an immunohistochemistry (IHC) staining assay of the kidney and liver tissues. As shown in Figure 6A,B, shikonin treatment could suppress TNFα, IL-6, and IL-1β protein levels in the kidney and liver tissues, similar to dexamethasone treatment. 

## 3. Discussion

Shikonin is a natural purple-red product extracted from *Lithospermum erythrorhizon* [35,36]. Recent pharmacological studies have shown that shikonin exerts anti-inflammatory, antitumor, bactericidal, and immune regulation effects [31,37,38,39]. For instance, 10 mg/kg per day of shikonin was found to suppress inflammation by modulating PI3K/Akt signaling pathway in a rat model of osteoarthritis by inhibiting TNF-α, IL-1β, and inducible nitric oxide synthase (iNOS) levels [40]. Moreover, shikonin can decrease the expression level of pro-inflammatory cytokines TNF-α, IL-6, and IL-12 to ameliorate isoproterenol (ISO)-induced cardiac injury in vivo [41]. Although the anti-inflammatory effects of shikonin on acute or chronic injury have been extensively reported, the relationship between shikonin and intestinal flora in terms of inducing anti-inflammatory effects remains poorly explored.

In recent years, gut flora has emerged as a critical mediator in the occurrence and progression of various diseases and pathologic phenomena. However, few studies have assessed the shikonin-induced effects on intestinal flora and the potential implications on acute inflammation. Accordingly, an in-depth exploration of the function of shikonin in intestinal flora could clarify its novel mechanism of action. Herein, we elucidated that shikonin administration could suppress mouse ear swelling in a model of acute inflammation when compared with the blank control treatment. Simultaneously, shikonin administration reduced the expression levels of inflammatory factors TNFα, IL-6, and IL-1β, indicating a potential anti-inflammatory effect. It is worth noting that shikonin and its derivatives have been reported to affect gut microbiota to potentially prevent colorectal cancer, with 20 mg/kg shikonin exhibiting the most effective preventive effects through the Wnt/β-catenin signaling pathway [31]. Moreover, other orally administered natural products or herbs have been shown to interact with the gut flora by modulating the composition or metabolism of intestinal flora. For example, cinnamon essential oil can directly suppress *Escherichia coli* and *Staphylococcus aureus*, thus altering the microbiota compositions and functioning as an antibiotic [42]. Importantly, the interactions between gut microbiota and natural products can help us better understand the mechanism of different diseases. However, several adverse features in vivo have been reported with shikonin, including its low water solubility, non-selective toxicity, and poor bioavailability, all of which could limit its pharmacological use [43,44,45]. Future investigations should pay considerable attention to improve its water solubility and bioavailability, which will broaden its applications without question.

Collectively, using 16S rDNA sequencing analysis, we found that the inhibitory effect of shikonin on inflammation involved intestinal flora, whose diversity, composition, and functions were altered following shikonin administration. Moreover, shikonin treatment could suppress the expression levels of inflammatory cytokines TNF-α, IL-6, and IL-1β, as determined via qPCR, Western blot, and IHC investigations. Therefore, we propose that shikonin may exhibit anti-inflammatory effects mediated via the gut microbiota to resist inflammation, markedly enriching our understanding of shikonin and providing an alternative mechanism for its anti-inflammatory role. 

## 4. Materials and Methods

### 4.1. Reagents and Antibodies

Shikonin (Meilunbio, S0921AS; purity, >98%) was occupied to treat the experimental group with 20 mg/kg for each mouse, while dexamethasone (Plant Chem Medicine company, Xi’an, China, PCM20356; purity, >98%) was used as positive control in the current study. Xylene (Macklin, X821391; purity, >99%) was utilized to induce a mouse model of acute ear edema. IL-6 (Cell signaling Technology, 12912), IL-1β (Bioteche, AF401-NA), and β-actin (ABclonal, AC026) antibodies were prepared for Western blot assay. 

### 4.2. Animal Breeding

A total of thirty-two healthy male C57BL/6 mice (age: 6 weeks old; weight: 20 ± 1 g) were purchased from the Experimental Animal Center of Fourth Military Medical University. All animals were raised under controlled conditions (at 22 ± 1 °C and 50 ± 10% humidity). After rearing them for one week, they were intragastrically administered soybean oil (100 μL/each), dexamethasone (20 mg/kg), and shikonin (10 or 20 mg/kg) every two days for two consecutive weeks. Dexamethasone and shikonin were dissolved in the soybean oil. Procedures were all under approval by the Institutional Review Board of the Fourth Military Medical University.

### 4.3. Mouse Feces Collection

To collect animal feces, the mouse was fixed on a clean white plate six hours after the last gavage on day 14. Then, we gently lifted its tail and pressed its lower abdomen with a finger to obtain fresh feces (3–5 pieces per tube) in sterile EP tubes with marked numbers. All collected feces were stored in −80 °C refrigerator for further 16S rDNA sequencing.

### 4.4. Animal Tools and Xylene-Induced Acute Ear Edema Model of Mice

The abovementioned mice were randomly divided into four groups (*n* = 8 for each group): the control group, positive control group with dexamethasone administration, and shikonin administration experimental group with 10 mg/kg low dosage and 20 mg/kg high dosage, respectively. 

On the last day (day 14) of intragastric administration, for each mouse, 50 μL of xylene was administered on the inner and outer sides of the right ear of each mouse to generate an acute inflammatory mouse model, while the left ear was untreated as a control. After 45 min, all the mice were sacrificed and the swelling rate was determined via cervical dislocation. A biopsy punch (diameter, 8 mm) on the ear piece was used to obtain samples for both ears. All ear samples were immediately weighed with a balance. The later results validated that 20 mg/kg of shikonin administration exhibited the higher anti-inflammation effect in the mouse models. Then, mice of the 20 mg/kg shikonin group were used to further investigate the alterations of intestinal microbiota by 16S rDNA sequencing.

### 4.5. Analysis of Mouse Ear Edema

The ear edema rate was measured via calculating the difference in weight between the left and right ears of the same mouse. The formulas are as follows:

Ear edema rate = (WR − WL)/WL × 100%; Inhibition rate = [(WR − WL)Ctrl − (WR − WL)Drug]/(WR − WL)Ctrl × 100%

where WR and WL are the weights of the right and left ears, respectively, and Ctrl subscripts the mean edema weight of the control group, while Drug subscripts the mean edema weight of the shikonin or dexamethasone group.

### 4.6. 16S rDNA Sequencing Analysis for Intestinal Flora

Genomic DNA of the feces was extracted firstly, and then, we monitored DNA concentration using 1% agarose gels. Next, 16S rDNA genes in distinct regions (16S V3-V4) were amplified by primers 515F (5′-GTGCCAGCMGCCGCGGTAA-3′) and 806R (5′-GGACTACHVGGGTWTCTAAT-3′) with Phusion^®^ High-Fidelity PCR Master Mix kit (New England Biolabs, Ipswich, Massachusetts, USA). Qiagen Gel Extraction Kit (Qiagen, Venlo, Netherlands) was utilized to purify the PCR products. Following the manufacturer’s guidelines, sequencing libraries were built via the NEBNext UltraTM II DNA Library Prep Kit (Cat No. E7645) and their quality was evaluated through a Qubit@ 4.0 Fluorometer (Thermo Scientific, Waltham, Massachusetts, USA) and Agilent Bioanalyzer 2100 system. Finally, by usage of Illumina NovaSeq platform, the conducted library was sequenced to gain 250 bp paired-end reads.

FLASH (Fast Length Adjustment of Short reads, Version 1.2.11, http://ccb.jhu.edu/software/FLASH/, accessed on 15 October 2022) software was used to merge the paired-end reads. To generate high-quality clean tags, fastp (version 0.20.0) software was performed to filter quality of the raw tags. Sequencing data were processed using the DADA2 methods (Divisive Amplicon Denoising Algorithm 2, version 1.16) and QIIME2 (Version QIIME2-202006) pipeline to obtain initial amplicon sequence variants (ASVs) with high resolution. 

Alpha diversity (the Chao 1, Shannon, dominance, and Simpson indices) was profiled to identify the diversity of the species of each sample, considering their content and evenness. For alpha-diversity measures, significant differences were assessed using the non-parametric pairwise Wilcoxon rank sum test. To assess the complexity of the species and compare the distinction between groups, beta diversity was assessed using the R software (Version 3.5.3). MetaStat and T-test analyses were carried out via R software to detect the species differences between groups at each taxonomic level (phylum, class, order, family, genus, and species). LEfSe analysis (LDA score threshold:4) was performed to evaluate the differentially abundant taxa using the LEfSe software (version 1.0). Heat maps were constructed at the genus level using the non-parametric Wilcoxon test (*p* < 0.05, q < 0.1).

### 4.7. RT-qPCR Assay

TRIzol reagent (Sangon Biotech, Shanghai, China, B511311-0100) was utilized to extract the total RNA. Then, a total of 1 μg RNA was transcribed into cDNA using PrimeScript RT Reagent Kit with gDNA Eraser (TaKaRa, Tokyo, Japan, RR047A) under the instructions. Then, qPCR assay was carried out through a FastStart Essential DNA Green Master kit (Roche, Basel, Switzerland, 06924204001) in accordance with the manufacturer’s procedures. The primers used in qPCR are listed in Table 1 below. Finally, the relative expression level of related genes was calculated using Ct values via the 2^−^^ΔΔCt^ method.

### 4.8. Western Blot Assay

Total proteins were extracted from liver and kidney tissues using radioimmunoprecipitation assay (RIPA) buffer. A BCA Protein Assay Kit (Thermo Scientific, Waltham, Massachusetts, USA) was bought to detect different protein concentrations. Then, the lysate samples were loaded on a sodium dodecyl sulfate (SDS)–polyacrylamide gel with an equal amount and moved onto polyvinylidene difluoride (PVDF) membranes (Millipore, Burlington, Massachusetts, USA). The immunoblots were incubated with primary antibodies against the inflammatory cytokines TNF-α, IL-6, IL-1β (1/1000 dilution), and β-actinin (1/5000 dilution) after the membrane was blocked by 5% milk and was then probed with horseradish peroxidase (HRP)-conjugated donkey anti-rabbit secondary antibodies (1/20,000 dilution) the next day. The immunoreactions were detected via an enhanced chemiluminescence (ECL) system in accordance with the manufacturer’s instructions. 

### 4.9. Immunohistochemical Assay

In the formal animal assay, kidney and liver tissues of mice in the three groups were collected to perform immunohistochemical assay. All tissues were immersed with 10% neutral formalin, and then embedded in paraffin. The sections were dehydrated, dewaxed and washed in PBS three times gently, and then added into 3% hydrogen peroxide solution in the dark for 30 min. Then, the tissues were blocked with 3% bovine serum albumin (BSA), treated with specific primary antibodies to incubate for overnight at 4 °C. Images were acquired using microscopy (Olympus BX53).

### 4.10. Statistical Analysis

Statistical analysis was performed using GraphPad Prism 9.0 (GraphPad Software, San Diego, CA, USA). Data were presented as the calculated mean ± standard deviation with triplicates unless otherwise noted. Significant differences between the two groups were analyzed using a two-tailed Student’s *t*-test and analysis of variance for the three groups (**** means *p* < 0.0001, *** means *p* < 0.001, ** means *p* < 0.01 and * means *p* < 0.05). Statistical significance was set at *p* < 0.05. The statistical analysis of 16S rDNA sequencing for the flora is outlined in this section.

## Figures and Tables

**Table 1:** Anti-inflammatory effects of shikonin by intragastric administration in vivo.

Compounds	Anti-Inflammatory Activity			
	Dose	Ear Edema Mean Weight ± SD (mg) ^a^	Inhibition Rate (%)	*p* Value ^b^
Control ^c^	100 μL soybean oil	13.01 ± 1.91	--	
Shikonin	20 mg/kg	10.12 ± 2.60	12.23	*
Dexamethasone ^d^	20 mg/kg	7.20 ± 1.24	12.46	*

Note: ^a^, anti-inflammatory inflammatory activity at 45min after xylene treatment; ^b^, * *p* < 0.05; ^c^, blank control group; ^d^, positive control group.

## Data Availability

The sequencing data generated in this study are accessible from the BioProject or SRA database (https://www.ncbi.nlm.nih.gov/sra/PRJNA892764, accessed on 21 October 2022) and the accession number is PRJNA892764.
